# Evolving Perspectives on Innate Immune Mechanisms of IPF

**DOI:** 10.3389/fmolb.2021.676569

**Published:** 2021-08-09

**Authors:** Genta Ishikawa, Angela Liu, Erica L. Herzog

**Affiliations:** ^1^ Section of Pulmonary, Critical Care and Sleep Medicine, Department of Internal Medicine, Yale School of Medicine, New Haven, CT, United States; ^2^ Department of Pathology, Yale School of Medicine, New Haven, CT, United States

**Keywords:** innate immunity, macrophage, pulmonary fibrosis, microenvironment, biomarker

## Abstract

While epithelial-fibroblast interactions are viewed as the primary drivers of Idiopathic Pulmonary Fibrosis (IPF), evidence gleaned from animal modeling and human studies implicates innate immunity as well. To provide perspective on this topic, this review synthesizes the available data regarding the complex role of innate immunity in IPF. The role of substances present in the fibrotic microenvironment including pathogen associated molecular patterns (PAMPs) derived from invading or commensal microbes, and danger associated molecular patterns (DAMPs) derived from injured cells and tissues will be discussed along with the proposed contribution of innate immune populations such as macrophages, neutrophils, fibrocytes, myeloid suppressor cells, and innate lymphoid cells. Each component will be considered in the context of its relationship to environmental and genetic factors, disease outcomes, and potential therapies. We conclude with discussion of unanswered questions and opportunities for future study in this area.

## Introduction

Idiopathic pulmonary fibrosis (IPF) is a progressive and incurable condition defined by the radiographic and histopathologic pattern of usual interstitial pneumonia (UIP) in the absence of an identifiable cause or exposure (American Thoracic Society, 2000; Raghu et al., 2011). With a 5 years survival rate of little more than 50%, it carries one of the worst prognosis of all interstitial lung diseases (ILDs) (Raghu et al., 2011). Numerous IPF risk factors have been identified including aging (Raghu et al., 2011), cigarette smoking (Baumgartner et al., 1997), chronic viral infections (Enomoto et al., 2003), gastroesophageal reflux (Raghu et al., 2006), and genetic predisposition (Hodgson et al., 2002), but the mechanisms through which these entities are related to the disease remain unknown. Thus, better understanding of fibrogenic processes affecting the lung remains a critical unmet need.

**FIGURE 1:**
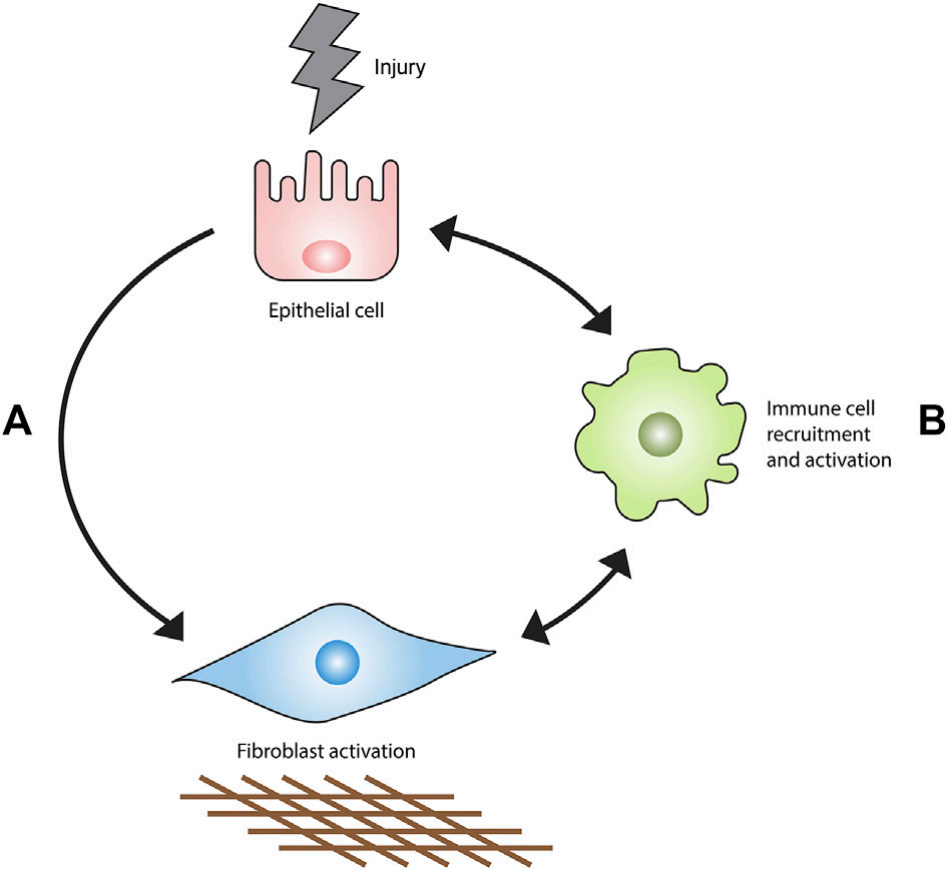
Current model of IPF pathogenesis. Epithelial injury results in fibrosis primarily through an interaction with fibroblasts **(A)** rather than the recruitment and activation of immune cell populations **(B)**. Immune cells accumulate in the injured lung and orchestrate the development, maintenance, progression or regression of fibrosis.

Parenchymal fibrosis is proposed to originate from prolonged or perpetuated alveolar epithelial injury (Fernandez and Eickelberg, 2012). This event stimulates an aberrant wound healing response characterized by myofibroblast expansion and the obliteration of lung tissue by excessive extracellular matrix (ECM) (Wynn, 2011). A substantial body of evidence generated from preclinical studies and clinical trials forms the basis for the current consensus that IPF does not appear to be a direct result of immune cell dysfunction but rather, that immune and inflammatory cells can permit, promote, or suppress fibrotic responses in the lung stroma (Figure 1). This article reviews the evidence in support of this hypothesis.

## Historical Perspective

The clinical features of IPF provide very limited insight into associated immune factors. Diagnostic criteria for IPF are essentially the presence of a pattern consistent with Usual Interstitial Pneumonia on chest imaging or lung biopsy in the absence of an identifiable cause. Compared to other ILDs, IPF lungs contains fewer inflammatory cells and chest imaging contains very little of the ground glass opacities that are typically reflective of inflammation (Raghu et al., 2011; Travis et al., 2013). Features of autoimmunity, such as physical exam findings and serology, as is clinical evidence of an identifiable antigen-driven immune response, are absent (Raghu et al., 2011). IPF outcomes are worsened by treatment with low dose Prednisone, Azathioprine (Idiopathic Pulmonary Fibrosis Clinical Research Network et al., 2012), and unaffected by treatment with biologics targeting interferon gamma (King et al., 2009) or TNFα (Raghu et al., 2008). These clinical observations have been interpreted by some sources as indicating the pathogenesis of IPF lacks an immune component (Blackwell et al., 2014). However, this array of findings is unlikely to suggest that the immune system is not involved. On the contrary, the worsening of clinical outcomes by classical immunosuppression suggests, if anything, that certain immune responses might be protective and others might be harmful. Several studies demonstrate that anti-fibrotic and immunomodulatory macrophage functions are suppressed in IPF, and classical immunosuppression might aggravate the loss of anti-fibrotic immune cells (van Geffen et al., 2021). Thus, better understanding of all forms of immunity has the potential to advance the understanding of IPF.

## Innate vs Adaptive Immunity

Immune responses are dichotomized into innate and adaptive processes. The former component, which is the focus of this review, is currently understood as a rapid and programmed response to chemical or physical stimuli and the latter being a specific response to a particular antigen. While abnormalities in both components have been described in IPF, the available evidence suggests a pathogenic contribution of innate immunity whereas the role of adaptive immunity is less clear. Thus, this review will focus on the innate immune processes that could contribute to IPF.

## Innate Immune Ligands in Idiopathic Pulmonary Fibrosis: Pathogen Associated Molecular Patterns vs. Danger Associated Molecular Patterns

Fibrotic immunopathology has been reported in the context of pattern recognition receptor (PRR) activation. PRRs respond to the physical or chemical structure of their respective ligands to initiate and in some cases perpetuate host defense. PRR-activating structures derived from microorganisms are termed “pathogen associated molecular patterns,” or “PAMPs” and substances generated by tissue injury are called “danger associated molecular patterns,” or “DAMPs” (Tang et al., 2012). Another type of PRR ligand, “homeostatic associated molecular patters,” or “HAMPs” has recently been described but has not yet been studied in IPF. It should be noted that while cells of the immune system and lung stroma contain functional PRRs, the focus of this review is restricted to professional immune cells involved in host defense (Mogensen, 2009). Thus, PAMPs, DAMPS, and PRRs will only be discussed in these contexts.

***Pathogen associated molecular patterns.*** While not viewed as an infectious process, several lines of evidence derived from animal and human studies support a connection between IPF and microbes. The contribution of viral and bacterial PAMPs has been explored as follows (Ellson et al., 2014).

***Viruses.*** IPF has been linked to a number of viruses. Several studies have shown that the Herpes virus family member Epstein-Barr virus is enriched in IPF bronchoalveolar lavage (BAL) fluid and lung tissues (Tang et al., 2001; Manika et al., 2007), and may contribute to induction of TGFβ1 expression and epithelial acquisition of mesenchymal properties (Sides et al., 2011). Likewise, Cytomegalovirus has been shown to exacerbate bleomycin induced fibrosis in mice through a mechanism involving canonical TGFβ1 activation and Vimentin expression (Li et al., 2016). A study in human lung epithelium revealed Herpesvirus saimiri infection promotes IL-17 production (Folcik et al., 2014). In line with these findings, antiviral therapy has shown modest benefit in animal models (Mora et al., 2007) and one small human study (Egan et al., 2011) but more widespread studies of efficacy and mechanism are currently lacking.

***Bacteria.*** Data linking bacteria to IPF have also been described. One study of IPF BAL revealed an inverse relationship between bacterial load and clinical outcomes where detection of organisms such as *Haemophilus*, *Streptococcus*, *Neisseria*, and *Veillonella* were predictive of lung function decline (Molyneaux et al., 2014). A separate study found that IPF BAL was enriched for strains of *Staphylococcus* and *Streptococcus* (Han et al., 2014), with the latter species aligning with increased nucleotide-binding oligomerization domain-like (NOD) receptor signaling and poor outcomes (Huang et al., 2017). While the reasons for these observations remains unclear, the known association of IPF with gastroesophageal reflux disease (GERD) (Lee et al., 2010), raises the possibility of chronic microaspiration as an inoculation source. Thus, the microbiome is emerging as a new area of study in IPF and has led to studies examining antibiotics as a novel treatment approach (Kawamura et al., 2017).

***Danger Associated Molecular Patterns*** Substances generated by tissue injury can function as danger associated molecular patterns, or “DAMPs.” Normal tissue turnover generates low levels of DAMPs which support homeostasis through the removal of debris, resolution of injury, and completion of repair (Ellson et al., 2014). DAMP overabundance, however, appears to favor a microenvironment characterized by PRR activation and inflammation (Land, 2015) that may emulate or differ from responses caused by PAMPs (Piccinini et al., 2016). PRR ligands generated by homeostatic mechanisms (homeostatic associated molecular patters, HAMPs) have also been described (Liston and Masters, 2017) but have not yet been studied in IPF.

Numerous substances can function as DAMPs. The easiest to conceptualize may be cellular components such as nucleic acid and organelles that are passively generated by the necroptotic lysis of dying/dead cells or actively released *via* exocytosis of membrane bound vesicles or endosomes. DAMPs are also generated by the cleavage of extracellular proteins into fragments that can act as signaling molecules. The PRRs that recognize DAMPs are, for the most part, also activated by PAMPs (Ellson et al., 2014) and in the setting of pulmonary fibrosis can be protective or pathogenic depending on the context. For example, absence of the dsRNA sensor toll-like receptor 3 (TLR3) worsens bleomycin induced pulmonary fibrosis in mice and humans possessing the Leu412 Phe polymorphism in the TLR3 gene develop a rapidly progressive form of IPF (O’Dwyer et al., 2013). TLR4 deficiency worsens, and TLR4 agonists improve, bleomycin induced fibrosis in mice *via* at least two mechanisms: augmentation of lung progenitor cell renewal (Liang et al., 2016) and modulation of TGFβ1 and IL-17 production (Liu et al., 2010). The role of TLR4 appears to be complex as its inhibition can also be protective (He et al., 2009; Li et al., 2015). A connection to IPF is suggested by the finding that endogenous TLR4 ligands such as high mobility group box 1 (HMGB1) (Park et al., 2004; Li et al., 2014), tenascin-C (Midwood et al., 2009; Carey et al., 2010; Bhattacharyya et al., 2016), S100 protein (Park et al., 2004) and hyaluronan fragments (Jiang et al., 2005) are enriched in IPF BAL and lung tissues (Huebener and Schwabe, 2013). Additional evidence linking TLR4 to IPF is the discovery mutations in the TLR2/4 adaptor protein Toll interacting protein (TOLLIP) increases disease susceptibility (Noth et al., 2013), though the mechanism of this association remains unknown. Intriguingly, N-acetylcysteine therapy is reported to be efficacious for individuals with IPF with an rs3750920 (TOLLIP) TT genotype (Oldham et al., 2015), therefore elucidating underlying biology of interaction between DAMPs and their receptors along with future clinical trials targeting these entities might lead to substantial clinical benefit.

The connection to innate immunity extends beyond Toll like receptors as shown by data implicating the NACHT, LLR and PYD domains-containing protein 3 (NALP3) inflammasome activation in IL-1β associated fibrosis in the bleomycin mouse model (Gasse et al., 2009; Tian et al., 2017). Recent work links this process to the mitochondrial antiviral signaling (MAVS) pathway in mice and humans with IPF (Kim et al., 2020). The inflammation may also be activated *via* toll-like receptor 9 (TLR9) (Trujillo et al., 2010) which along with its endogenous ligand mitochondrial DNA (mtDNA) is increased in IPF (Ryu et al., 2017). In fact, previously normal lung fibroblasts stimulated with TLR9 agonists develop features of myofibroblasts (Kirillov et al., 2015; Ryu et al., 2017) and excessive concentrations of circulating mtDNA is predictive of poor outcomes in several IPF cohorts (Ryu et al., 2017; Sakamoto et al., 2021). The inflammatory nature of the bleomycin model has made this biology difficult to understand (Luckhardt et al., 2011), heightening the need for experimental systems that are more reflective of the healthy and diseased human lung. Finally, abnormal responses to DAMP recognition have been described as one form of immunosenescence (Huang et al., 2015) and it is intriguing to consider this concept in relationship to the telomerase mutations that are associated with the IPF disease state. Thus, the connection of PAMPs, DAMPs, and PRRs in IPF is an area warranting further studies.

## Innate Immune Cells

As the first line of defense against invading pathogens, cells of the innate immune system have important roles in host defense and tissue homeostasis. The best studied cells in the context of IPF are macrophages and neutrophils, though more recently described populations such as fibrocytes, myeloid-derived suppressor cells (MDSCs), and innate lymphoid cells (ILCs), may also be involved. It is worth noting that stromal populations such as fibroblasts and epithelium also demonstrate innate immune functions (Selman and Pardo, 2006) but because these cells are not professional immune cells, their at best speculative contribution to IPF immune dysfunction will not be discussed herein.

***Macrophages*** are both the major antimicrobial phagocytes in the lungs and central mediators of fibrotic lung disease (Wynn and Vannella, 2016). Macrophages can regulate both injury and repair in various models of fibrosis and macrophage driven processes have been important areas of study in IPF for more than 50 years (Bitterman et al., 1986; Blackwell et al., 2014). Older paradigms proposed dichotomized phenotypic categories of classically activated macrophages, or “M1s” generated by INFγ and TNFα exposure as being antifibrotic, and alternatively activated macrophages or “M2s” generated by IL-4, IL-10, IL-13, and TGFβ1 stimulation as being profibrotic (Pechkovsky et al., 2010; Wynn and Vannella, 2016). However, as macrophage classification schemes have become increasingly nonbinary, the M1/M2 dichotomy may be grossly oversimplified (Gordon et al., 2014) yet conceptually useful when describing the functional characteristics of these adaptable cells. Specifically, in this context, a moderate excess of M1-like macrophages suppresses fibroblast activation and ECM accumulation, while a large excess may cause epithelial cell death and diffuse alveolar damage similar to that occurring in acute exacerbations of IPF. Similarly, a controlled balance of M2 macrophages stimulates appropriate repair and regeneration while an excess of M2 macrophages drives the lung towards progressive and inexorable fibrosis (Zhou et al., 2014). Animal modeling of IPF reveals that the plasticity and diversity of lung macrophages involves contributions from long lived, lung resident alveolar macrophages (Larson-Casey et al., 2016) and from populations of interstitial macrophages arriving from the bone marrow and circulation (Peng et al., 2016). In humans, accumulating evidence suggests that an increase in circulating monocytes predicts poor outcomes, as one of the most validated cellular biomarkers in IPF (Scott et al., 2019) though it is currently unknown whether these findings relate to a primary hematopoietic defect or are simply undergoing appropriate recruitment to the injured and fibrotic lung. Either way, because monocytes presumably give rise to interstitial macrophages these observations could be used to develop cellular biomarkers reflective of pathogenesis and outcome. While differences in polarization markers precludes direct comparison of mouse and human macrophages, the preponderance of available evidence indicates that expression of scavenger receptors and profibrotic markers is a cross-species feature of many forms of lung fibrosis including IPF (Christmann et al., 2014; Zhou et al., 2014; Zhou et al., 2015) though again, why these cells are aberrantly polarized and whether they promote disease in humans remains unknown.

Macrophages are implicated in fibrotic processes *via* a large number of mechanisms, none of which involve direct production of extracellular matrix. Since the 1980s, alveolar macrophages from IPF patients have been known to modulate fibroblast activation *via* the production of mediators that have come to be associated with M2 activation. At the time that these studies were first performed, the concept of innate immunity had not yet been established and these observations were viewed as IPF being an inflammatory condition though this idea is being continuously refined and reimagined as the field evolves. More recent studies have shown that interstitial macrophages from mice and humans display fibrosis promoting properties (Zhou et al., 2014), as do circulating monocytes isolated from patients with IPF (Zhou et al., 2014). The latter finding is notable for demonstrating that monocytes are programmed to promote fibrosis before entering the lung. Mouse modeling has shown that removal (Gibbons et al., 2011; Murray et al., 2011) or repolarizing (Murray et al., 2011) of lung macrophages can prevent and reverse experimentally induced mouse fibrosis. The repolarization hypothesis forms the basis for the use of the evolutionarily conserved pattern recognition protein pentraxin 2, which is under investigation as a novel therapeutic in several fibrotic diseases including IPF (Murray et al., 2010; Murray et al., 2011).

**FIGURE 2:**
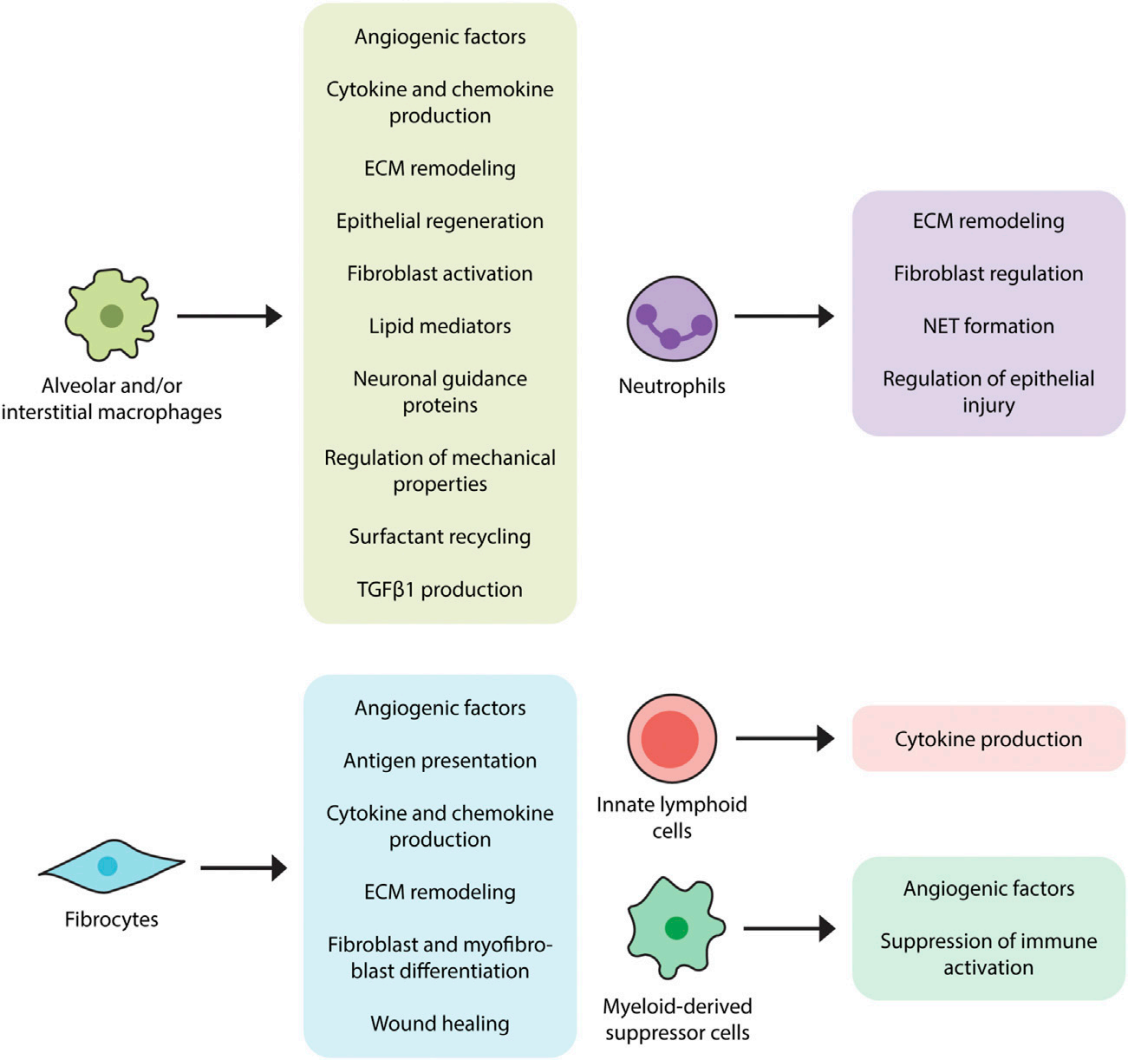
Proposed contributions of innate immunity in pulmonary fibrosis. Innate immune cell populations, ranging from macrophages to innate lymphoid cells, have displayed fibromodulatory properties in response to stimuli such as pathogen-associated molecular patterns (PAMPs) and danger-associated molecular patterns (DAMPs). Alveolar and interstitial macrophages produce TGFβ1, angiogenic factors, various cytokines and chemokines, lipid mediators, and neuronal guidance proteins, induce ECM remodeling, and regulate surfactant recycling and mechanical properties of the lung. Neutrophils also participate in the regulation of ECM remodeling, as well as the formation of extracellular neutrophil traps, which may have pro-fibrotic effects. Fibrocytes, in accordance with their multilineage features, exhibit a variety of fibrosis-promoting functions, including antigen presentation, cytokine and chemokine production, differentiation into fibroblasts and myofibroblasts, and wound contraction. Myeloid-derived suppressor cells (MDSCs) function to dampen immune responses and are also involved in vascular remodeling while innate lymphoid cells (ILCs) have been shown to produce pro-fibrotic cytokines.

These contributions are accompanied by additional mechanisms (Wynn and Vannella, 2016), some of which include interactions with dead or dying cells (Ellson et al., 2014). For example, efferocytosis (engulfment of apoptotic cells) induces transcriptional activation of *Tgfb1* in alveolar and interstitial macrophages (Freire-de-Lima et al., 2006) which is in line with a well-established literature implicating alveolar macrophages and/or LysM + cells as a source of TGFβ1 in humans (Toossi et al., 1996) and mice (Young et al., 2016). The functions of apoptotic cell clearance and TGFβ1 production are augmented by production of cytokines (TNFα, IL-1, IL-6, IL-8, IL-10, and IL-12) and chemokines such as CXCL1, CXCL2, CXCL9, CXCL10, CXCL12, CCL5, CCL17, and CCL18 (Arango Duque and Descoteaux, 2014). Through production of lipid mediators such as eicosanoids they might contribute to fibrosis (Huang and Peters-Golden, 2008), though this function has yet to be confirmed in IPF tissues and experimental modeling (Castelino, 2012). Additional functions include ECM remodeling and matrix metalloproteinase production (Rohani et al., 2015) as well as the ingestion and recycling of collagen (Madsen et al., 2013). While several studies indicate that macrophages might also contribute to pulmonary fibrosis by regulating epithelial cell activation (Young et al., 2016), this area remains largely unexplored in the context of IPF. Macrophages are also known to direct (Biswas and Mantovani, 2012) and respond to the metabolic products of adjacent cells (Biswas and Mantovani, 2012; Xie et al., 2015) and because they recycle surfactant (Wright, 1990), they may be involved in the poorly understood recycling association between surfactant protein mutations and IPF (Lawson et al., 2004). Macrophages regulate the expression of pro- and anti-fibrotic angiogenic factors such as vascular endothelial growth factor (VEGF) (Stockmann et al., 2010), which may be relevant given the efficacy of therapies targeting vascular endothelial growth factor 2 (VEGFR2) in IPF (Barratt et al., 2018). Macrophages express neuronal guidance proteins such as Netrin-1 which appears to control a newly recognized form of adrenergic nerve associated fibrosis in mouse models and in patients with IPF (Gao et al., 2021). Macrophages show a connection to epithelial regeneration through their production of WNT-containing exosomes (Saha et al., 2016) which may also be implicated in the poorly understood association between innate immune activation and lung progenitor cell survival (Liang et al., 2016). Finally, macrophages both contribute to (Solis et al., 2019) and respond to altered mechanical properties in the fibrotic lung, suggesting an immunomechanical function (Solis et al., 2019). The fibrosis promoting functions of lung macrophages are depicted in Figure 2.

The compelling connection between macrophages and fibrosis has fostered the development of immune targeted therapies in IPF. To date, most clinical trials in this arena examining a specific mediator have failed to show a benefit of this strategy. Specifically, TNFα neutralization with Etanercept, which would be expected to suppress M1 activation, did not affect outcomes in a Phase III trial (Raghu et al., 2008). An approach targeting macrophage recruitment with a monoclonal antibody targeting chemokine C-C chemokine ligand 2 (CCL2) was similarly unsuccessful (Raghu et al., 2015). Additionally, despite early promise, administration of recombinant interferon gamma was also abandoned due to lack of efficacy (Raghu et al., 2004). While disappointing in terms of efficacy, these studies were useful in revealing that inhibition of one specific macrophage mediator or function is insufficient in delaying disease progression. The multiple combined body of work suggests that successful targeting of macrophages may require a more pleiotropic approach activation state and might be more efficacious. This hypothesis forms the rationale for the recently initiated Phase III trial of recombinant Pentraxin 2, an acute phase reactant that interferes with innate immune activation by binding to debris and inhibiting Fcγ receptor driven inflammatory process in phagocytic cells (Castano et al., 2009).

***Neutrophils*** are another innate immune population which may impact IPF in several ways. Early studies of BAL neutrophilia identified in a subgroup of IPF patients experiencing reduced survival (Kinder et al., 2008) and the neutrophil chemoattractant, CXCL8, is increased in IPF (Xaubet et al., 1998) suggesting an association between these entities. Additionally, BAL neutrophils are directly proportional to concentrations of an alveolar epithelial marker, cytokeratin 19, which further supports a connection in this regard (Inage et al., 2000). While these studies are most easily interpreted as supporting fibrosis as a neutrophil driven process, an alternate explanation is that neutrophils are in fact protective and that their accumulation represent the host’s attempt at restoring homeostasis. This hypothesis would be supported by observations made in other modeling systems in which neutrophils were found to suppress alveolar injury through their transfer of miR223 containing extracellular vesicles (Neudecker et al., 2017). However, because this function has not been shown in IPF, its relevance in this condition and related processes is at best uncertain.

Neutrophils also play an important role in ECM turnover. Specifically, the most well studied proteolytic product of alveolar neutrophils, neutrophil elastase (NE), is increased in IPF BAL (Obayashi et al., 1997). The involvement of NE in the generation of DAMPs and ECM degradation (Hedstrom, 2002; Chua et al., 2007) suggests one function for this enzyme. Furthermore, NE itself may regulate fibroblast responses in a manner that does not involve ECM breakdown (Gregory et al., 2015). When viewed in this light, it is notable that mice deficient in NE are protected from fibrosis in several lung models (Chua et al., 2007; Gregory et al., 2015) and that the NE inhibitor, Sivelestat, dampens fibrosis in the bleomycin model (Takemasa et al., 2012). Neutrophils also participate in by controlling the balance of TIMPs and MMPs (Beeh et al., 2003; Kruger et al., 2015) such as the pro-fibrotic MMP-2, MMP-8, and MMP-9 (Corbel et al., 2001; Henry et al., 2002), though the relevance of these findings to human IPF remains unclear.

Finally, an additional fibrogenic neutrophil function is the formation of extracellular neutrophil traps. These highly inflammatory aggregates of chromatin and neutrophils regulate activation of immune cells (Kaplan and Radic, 2012) and fibroblasts (Chrysanthopoulou et al., 2014). Detection of neutrophil extracellular traps (NETs) has been reported in the lungs of bleomycin exposed mice and in some forms of fibrotic ILD but not, as yet, in IPF (Chrysanthopoulou et al., 2014). Further studies are warranted to understand whether NETs play a role in IPF pathogenesis.

To summarize, neutrophils could participate in IPF through their production of soluble mediators, regulation of epithelial injury, control of ECM turnover, and formation of NETs. (Figure 2). However, because IPF lung tissue contains few neutrophils their role in this disease state remains unclear.

***Fibrocytes*** are bone marrow-derived cells displaying characteristics of leukocytes, hematopoietic progenitor cells, and fibroblasts. Viewed as originating from monocytes, they are found in the systemic circulation from which they migrate to areas of tissue injury and participate in wound repair (Strieter et al., 2009). Fibrocytes are associated with conditions characterized by chronic inflammation and fibroblast-mediated tissue remodeling such as IPF (Reilkoff et al., 2011) and related conditions affecting the lung (Odackal et al., 2020) and other organs (Reilkoff et al., 2011). They can be identified through their multilineage features ranging from cell surface markers to the production of various extracellular matrix components (Gomperts and Strieter, 2007; Strieter et al., 2009). Their mesenchymal properties are further reflected by their potential for differentiation into myofibroblasts with subsequent αSMA expression and involvement in wound healing (Hong et al., 2007; Herzog and Bucala, 2010; Kao et al., 2011). Fibrocytes have also been shown to influence fibrosis progression in mouse models through mechanisms involving paracrine regulation of fibroblast proliferation and activation (Madala et al., 2014) and augmenting accumulation of WT-1 positive mesenchymal cells in severely fibrotic lung regions (Sontake et al., 2015). Immunomodulatory functions of fibrocytes include expression of chemokine receptors such as CCR3, CCR5, CCR7, and CXCR4 which likely participate in their migration to areas of tissue injury. Fibrocytes might also contribute to a pro-inflammatory microenvironment by producing soluble mediators such as cytokines (IL-1β, IL-6, IL-10, and TNF-α) and chemokines (MIP-1α, MIP-1β, MCP-1, IL-8, and GRO-α). They can also initiate adaptive immune responses by presenting antigens to T helper cells (Chesney et al., 1997; Chesney et al., 1998; Quan et al., 2006; Fan and Liang, 2010). Although current technical challenges involving both detection and reproducibility limit their use in the clinical setting (Hu et al., 2015), fibrocytes may serve as both therapeutic target and predictor of poor outcome (Griffiths et al., 2018). Other areas of uncertainty include whether fibrocytes are a unique leukocyte population or are instead a subset of inflammatory monocytes characterized by collagen production (Etich et al., 2019), and whether the increase in circulating fibrocytes reported in IPF and related conditions indicates specific expansion of these cells or merely reflects the monocytosis described earlier in this article. Given their functional association with fibrogenesis, the specific role of fibrocytes in IPF pathogenesis is an area of interest that would benefit from further investigation. The fibrosis promoting functions of fibrocytes are depicted in Figure 2.

***Myeloid-derived suppressor cells*** (MDSCs) are a heterogenous population of myeloid origin first observed in cancer patients. Despite their reported heterogeneity, MDSCs share the common function of contributing to regulatory T cell (Treg) expansion and, subsequently, suppressing T cell activation and proliferation. Pathologically, there is an increasing evidence of MDSCs being involved in non-malignant inflammatory diseases including fibrotic disorders (Gabrilovich and Nagaraj, 2009; Lindau et al., 2013; Zoso et al., 2014). Specific to IPF, at least one study has shown that enrichment of monocytic MDSCs in the peripheral blood of IPF patients correlates with worsened lung function in those patients (Fernandez et al., 2016). Along those lines, MDSCs have also been associated with severe pulmonary hypertension, a well-known complication of IPF, in the bleomycin mice model with attenuation of the diseased condition achieved through chemokine receptor inhibition reducing MDSC recruitment (Bryant et al., 2018). The vascular remodeling observed in this complication may not only be a byproduct of fibrotic disease but has also been suggested to play a role in the progression of fibrosis through the mediation of epithelial injury and repair (Murray et al., 2017). The recruitment and involvement of MDSCs in the creation of a pro-fibrotic, immune dysregulated environment indicates that they may be a target of interest for therapies aiming to mitigate the development and progression of IPF and, as such, should be studied further in human IPF cohorts (Figure 2).

***Innate Lymphoid Cells*** are recently identified lymphoid cell populations distinguished by their lack of recombination activating gene (RAG) and classical T or B cell receptors (Bando and Colonna, 2016). The lack of RAG and classical receptors implies that their immunomodulatory functions arise from their response to intrinsic innate immune stimuli rather than the specific epitope driven activation that characterizes most other lymphocytes. ILCs contain at least three subgroups: ILC1, including IFN-γ-producing natural killer cells (Kiessling et al., 1975); ILC2, including a population of cells producing the Th2 cytokines IL-5 and IL-13; and ILC3s, including cells that produce IL-17 and IL-22 (Sonnenberg and Artis, 2015). ILCs in the lung form an immune system network in the lung by interacting with epithelial cells, natural killer T cells and myeloid (Lai et al., 2016). In the context of fibrosis, ILC2 respond to antigens and pathogens by releasing large quantities of IL-13 which makes them attractive targets in pulmonary fibrosis (Peebles, 2015). While ILCs have been identified in lungs of patients with IPF (Monticelli et al., 2011), their disease contribution is at best nascent and would benefit from additional investigation. The potential role of ILCs in fibrosis is shown in Figure 2.

## Synthesis and Summary

The connection between innate immunity and IPF continues to evolve and now encompasses a contribution from numerous processes and cell populations enacting competing and overlapping functions. The presence of these processes in fibrotic conditions affecting numerous organs frames innate immune dysfunction as a convergent molecular feature of divergent clinical states. The detection of these mediators in both diseased organs and the systemic circulation could represent a more significant hematologic contribution than previously believed. Conversely, it could represent the nonspecific and intrinsic nature of the response. When compared with epithelial cells and fibroblasts, whose proposed role in fibrosis is relatively well defined, the more heterogeneous contribution of innate immunity is nuanced and unlikely to respond to a single intervention which may make it challenging to target. Additional challenges include critical aspects of host defense and tissue homeostasis both in the lung and in distant organs. Because some of these limitations may be overcome by the relative ease of isolating immune cells and mediators from bronchoalveolar lavage and blood, innate immunity is an attractive area for the development of personalized therapies based on easily accessible biomarkers. Areas of particular interest and important questions in this context that would benefit from concerted efforts performed in large scale multicenter recruitment efforts, leveraging of existing datasets and registries, and the generation of improved modeling systems that more faithfully recapitulate the complex microenvironment of the fibrotic human lung and improve the understanding and treatment of IPF on a global scale are shown in Box 1. Better understanding of innate immunity will continue to shape our view of this disease and provide the potential for paradigm shifts in treatment and management.

Box 1 **Unanswered questions Regarding the Innate Immune System in IPF**

Is innate immunity protective or pathogenic in IPF?

Are PRRs that recognize DAMPs or PAMPs protective or pathogenic in the setting of pulmonary fibrosis?

Does the altered microbiome cause PAMP driven innate immune activation in IPF?

Does perpetuated microinjury cause DAMP driven innate immune activation in IPF and are therapies targeting DAMPs and their receptors efficacious in IPF?

Can immune events detected in the circulation be used to guide personalized therapies in IPF?

Do macrophages participate in pulmonary fibrosis *via* the regulation of epithelial cell activation?

Does production of lipid mediators in macrophages contribute to pulmonary fibrosis?

Can therapies targeting macrophage activation stabilize or restore lung function in patients with IPF?

Are NETS an important part of IPF pathogenesis?

Can fibrocytes be a therapeutic target or biomarker in IPF?

Do myeloid-derived suppressor cells or ILCs participate in IPF?

How do circulating myeloid cells, other than monocytes, contribute to the increased pool in the lung? Do they invade the lung and provide “transitory or permanent” populations *in situ*?
